# Size discrimination in adult zebrafish (*Danio rerio*): Normative data and individual variation

**DOI:** 10.1038/s41598-020-57813-1

**Published:** 2020-01-24

**Authors:** Maria Santacà, Tiziano Caja, Maria Elena Miletto Petrazzini, Christian Agrillo, Angelo Bisazza

**Affiliations:** 1 Department of General Psychology, University of Padova, Padova, Italy; 2 Department of Life Sciences and Systems Biology, University of Torino, Torino, Italy; 3 School of Biological and Chemical Sciences, Queen Mary University of London, London, UK; 4 Padova Neuroscience Center, University of Padova, Padova, Italy

**Keywords:** Perception, Object vision

## Abstract

In humans, aging and neurodegenerative diseases have been found to be associated with impairment in both mathematical abilities and estimation of continuous quantities such as size, weight or distance. Zebrafish (*Danio rerio*) is rapidly becoming a model for human aging and brain disorders but we currently lack any instrument for rapid assessment of quantity estimation abilities in this species. Here we developed a simple method based on spontaneous preference of zebrafish for using the larger available hole to pass an obstacle. We collected a large amount of data from small groups of zebrafish moving between compartments of their tank and we used these normative data to compare the performance of individually tested fish. Zebrafish significantly discriminated size ratios from 0.60 to 0.91 with their performance decreasing while increasing the size ratio between the smaller and the larger hole presented. On average, individually tested fish showed the same performance, but a large inter-individual variability was observed. Test-retest analyses revealed a good reliability of this test, with 0.60 and 0.75 ratios being the most informative. Experience did not affect individual performance, suggesting the suitability of this test to measure the longitudinal changes and the effects of pharmacological treatments on cognitive abilities.

## Introduction

Numerous neuropathologies such as some forms of dementia and some psychiatric disorders are associated with a decline in quantity estimation performance. Such decline can affect both numerosity estimation (e.g.^1,2^) and the estimation of continuous quantities such as object size, weight and distance or the duration of an event (e.g.^3–5^). Both deficits are often used for the differential diagnosis of some neuropathologies (e.g.^6,7^). In some cases, the estimations of continuous quantities can be compromised already in the early stages of the disease and, therefore, could potentially be used for an early diagnosis (e.g.^4−8^).

Both continuous quantity and numerical abilities have been used to investigate cognitive decline in animal models of normal and pathological aging. For example, young dogs (*Canis familiaris*) were found to learn faster than senior dogs to discriminate between objects of different size and were more efficient in transferring this knowledge to successive tests^9^. Another study found that deficits in size discrimination were associated with β-amyloid accumulation in the entorhinal cortex in aging dogs^10^. In the Western lowland gorilla (*Gorilla gorilla gorilla)*, older individuals were slower and less accurate than younger individuals in numerical discrimination and summation^11^.

Zebrafish (*Danio rerio*) are increasingly used as a model in biomedical research, including for research on human neuropathologies, due to its amenability to genetic manipulation, high-resolution imaging and to high throughput *in vivo* screening. Different zebrafish lines have been generated with alterations of TAU protein functioning that induce early neuronal disturbances and cell death. The alterations resemble the key pathological features of human TAU-related pathologies^12^. Zebrafish have also been used to examine the neurodevelopmental basis of psychiatric disorders and develop new antipsychotic drugs (e.g.^13,14^), and to model remyelination process in pathologies as multiple sclerosis or brain injuries^15^.

To facilitate the use of zebrafish as a model to explore age-related neuropathologies (where learning difficulties and disruption of size discrimination are early features in humans) we aimed to develop a new test to evaluate easily and relatively quickly the ability of size estimation in fish. There has recently been a growing interest in studying discrimination abilities in fish. Numerical abilities have been thoroughly investigated in various species (reviewed in^16,17^) including some studies of zebrafish (^18,19^). By contrast, estimation of continuous quantities has received relatively less attention. However, mosquitofish were found to be quite accurate in selecting the larger of two available mates, as well as the larger of two bidimensional figures, and guppies were found to be more precise in discriminating the size of individual food items than their numerosity^20–22^. Commonly these studies are based on associative learning or required complicated setup and procedures. In general, the methods so far developed require a considerable investment of time and work and are not ideal for large-scale studies (reviewed in^17,23,24^).

In a recent study we observed that fish have a natural tendency to pass through the larger of two available holes to move in their environment (Caja T, unpublished Master’s thesis, 2019). To assess normative species-specific ability of size discrimination, in the first experiment we recorded the behaviour of small groups of resident adult zebrafish while moving between the two halves of their home tank through pairs of holes whose size differed to a variable ratio. In the second experiment, we focused on individual variation applying the same type of test to individual subjects tested three times at weekly intervals. We finally investigated if previous experience influenced the ability to perceive and discriminate different sizes.

## Methods

### Experiment 1: Group quantity discrimination abilities

#### Subjects

We tested six groups of six naïve adult female zebrafish, *Danio rerio*, making a total of thirty-six fish that were 6 months old. Only females were used in this experiment as in mixed groups male court females and in male-only groups intra-sexual aggression often occurs. They originated from a large outbred stock (>200 adults) maintained in our laboratory and derived from adult zebrafish bought from a local supplier. From hatching to approx. 40 days, larvae were housed in petri dishes and then transferred in 80 l aquaria with gravel and vegetation. At 10–12 weeks fish were moved to 400-litre plastic tanks provided with natural gravel bottom, plants and two biomechanical filters. Water temperature was maintained at 26 °C and a 30 W fluorescent lamp illuminated each tank according to a 12 h:12 h light/dark photoperiod. Adult zebrafish were fed with commercial food flakes (Aqua tropical, Padovan®) in the morning and live brine shrimps, *Artemia salina*, in the afternoon.

#### Apparatus

**Figure 1:**
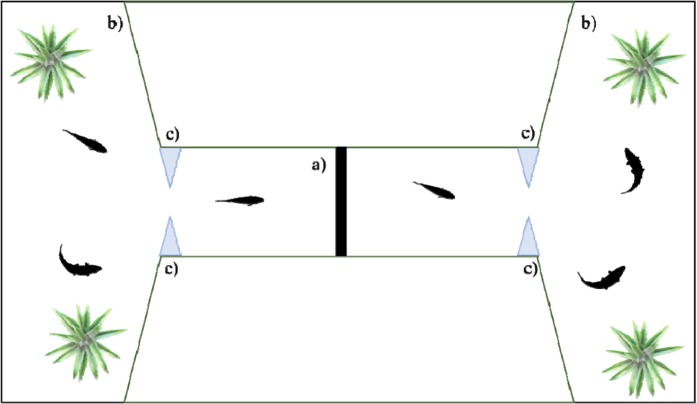
Experimental tank of Experiment 1. The apparatus was composed of a test panel (a) in the middle of the corridor, a frontal and a posterior compartment with natural plants and four acetate triangular prims at the ends of the central corridor (**c**).

The experiment was conducted in an 80 × 40 × 38 cm glass tank filled with 30 cm of water that was shaped as an hourglass by means of green plastic partitions (Fig. 1). A frontal and a posterior compartment were connected through a central corridor (28 × 13 cm) and were provided with plants (Fig. 1). The experimental tank was also provided with loose gravel to create a naturalistic environment. All the sides of the tank were covered with green plastic to isolate the fish from the outside environment to avoid any influence. One 30-W fluorescent lamp was placed above the frontal compartment, and one above the posterior compartment. At the ends of the corridor, four acetate triangular prims allowed the subject to centrally enter in the corridor (Fig. 1). In the middle of the corridor, they could perform the task spontaneously passing through one of two holes of a plastic panel (10 × 9 cm; Fig. 1) that was 3D printed with black PLA material. In this way, fish could move from one side to the other side of the tank. We used two identical apparatuses at the same time, both in the same dark room. One video camera was placed above the central corridor of each tank.

#### Procedure and stimuli

**Figure 2:**
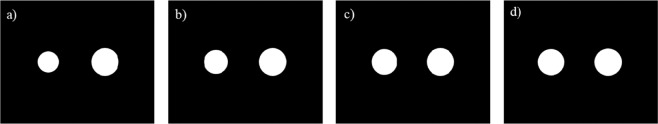
Experimental panels of Experiment 1. Subjects were presented with four different ratios: ratio 0.60 (**a**), ratio 0.75 (**b**), ratio 0.86 (**c**) and ratio 0.91 (**d**).

Six zebrafish were inserted at the same time in each experimental tank. For two days, a black panel with one central hole was inserted in the tank. In this phase we used a large hole (2.7 cm in diameter) to facilitate habituation of subjects to swim through the hole and to move between compartments. On the third day, this panel was substituted with similar panels provided with two holes of different size. In the first experiment, the test phase lasted six consecutive days during which we randomly alternated the panels with four types of size discrimination trials: ratio 0.60, ratio 0.75, ratio 0.86 and ratio 0.91 between the areas of the two holes (Fig. 2). Hole diameters ranged between 1.37 and 1.8 cm (see Table 1 for details). They were all larger than the minimum hole through which an adult zebrafish passes (0.8 cm in diameter), as determined in a preliminary trial. Subjects were recorded for three hours in the morning and three hours in the afternoon for a total of six hours each day with an hour interval in which no panel was present in the tank. Each ratio was presented for a total of 9 hours subdivided in six observations of 1.5 hour each; in half of them the bigger hole was presented on the frontal right side of the tank and in the other half on the frontal left side to avoid any side bias.

**Table 1:** Hole diameters for the four ratios used in Experiment 1.

Diameter of larger hole	Diameter of smaller hole	Ratio between the diameters	Ratio between the areas
1.8 cm	1.39 cm	**0.77**	**0.60**
1.8 cm	1.56 cm	**0.87**	**0.75**
1.8 cm	1.67 cm	**0.93**	**0.86**
1.8 cm	1.72 cm	**0.96**	**0.91**

#### Control Experiment A: the effect of the size of the habituation hole

The possibility exists that the habituation with a large hole (2.7 cm in diameter) could have affected the performances of the subsequent experimental trials. Indeed, preference for the larger hole during the test could be a consequence of learning and generalization to pass through a big hole rather than the consequence of a spontaneous preference for the bigger of two holes. To rule out this possibility, we performed a control experiment replicating Experiment 1 with the sole difference that in the habituation phase we used a hole of 1.0 cm, smaller than the smallest hole presented in the test phase (1.39 cm). Overall, we tested 4 groups of 6 naïve female zebrafish with this procedure.

If the subjects generalized learning that occurred in the habituation phase, they were expected to significantly prefer the smaller holes in the test phase. On the contrary, if the diameter of the hole used during the habituation was irrelevant, we expected a preference for the larger hole as observed in Experiment 1.

#### Control Experiment B: the effect of the absolute size of the test holes

In Experiment 1 as the ratio between holes increases, the average absolute size of the holes also increases. As the two dimensions co-vary, it is difficult to sort apart the influence of size ratio and absolute size. In this control experiment, we tested zebrafish with the same procedure of the first experiment, but here we systematically varied the size of the two holes while ratio was kept constant. We presented four different pairs of holes. The ranges of the four versions partially overlap and therefore the same hole could be the larger in one pair and the smaller in another (see Table 2). Varying the absolute size, we also partially controlled for the fact that in Experiment 1, during the testing phase, subjects had substantially more exposure to the larger hole (as the ratio was always adjusted by varying the size of the smaller hole). Overall, we tested 4 groups of 6 naïve female zebrafish with this procedure. If the absolute size of the holes was irrelevant, we expected no difference among the four pairs presented.

**Table 2:** Hole diameters for the four versions used in Control Experiment B.

Version	Diameter of larger hole	Diameter of smaller hole	Ratio between the diameters	Ratio between the areas
Version 1	1.90 cm	1.65 cm	**0.87**	**0.75**
Version 2	1.77 cm	1.53 cm	**0.87**	**0.75**
Version 3	1.63 cm	1.41 cm	**0.87**	**0.75**
Version 4	1.50 cm	1.30 cm	**0.87**	**0.75**

#### Data collection and statistical analyses

Experiment 1. From the video recordings, we scored the number of passages through each hole for every panel presented. One-third of the videos of each group were analyzed by two different experimenters to assess interrater reliability. Analyses were performed in R version 3.4.2 (The R Foundation for Statistical Computing, Vienna, Austria, http://www.r-project.org). Each group of six fish was considered as one datapoint with no distinction between the six subjects. However, for all six groups we checked that every subject participated in the experiment. To do this, in each group we randomly selected six intervals of 30 minutes, one for each ratio. Then we analysed 6 times the same video, following each time a different subject. We did not find any significant difference between the individual proportions of passages (all *P*-values > 0.746). We used binomial tests to compare the passages through the bigger hole in every ratio with chance level. A linear mixed-effects model (LMM, ‘lmer’ function of the ‘lme4’ R package) was performed to compare the total number of passages between the four ratios. To compare the performance (passages through the bigger hole) between the different ratios of the first experiment and the effect of the day, we used a LMM fitted with group as random effect. Subsequently, all pairwise comparisons were performed with Tukey post-hoc tests. To assess potential differences between groups, we performed a test of the random-effect term of the LMM (‘ranova’ function of the ‘lmerTest’ R package).

Control Experiment A. Data collection was performed identically to Experiment 1. Performances (passages through the bigger hole) in relation to the different ratios were analysed as in Experiment 1. A second LMM was conducted on the pooled data of Experiment 1 and Control Experiment A, adding diameter of the habituation hole as a factor.

Control Experiment B. Data collection was performed identically to Experiment 1. Performances (passages through the bigger hole) in relation to the different absolute diameter were analysed as in Experiment 1. A second LMM was conducted comparing the four versions of holes of this experiment with the data of the same ratio in Experiment 1.

Bayesian tests. The null hypothesis testing approach is powerful at detecting differences but does not allow testing for similarity between samples; in fact, the absence of a significant effect could be ascribed to several different factors, such as a low sample size^25^. In the case of a lack of significant effect in the control experiment, we used Bayesian tests that allowed us to directly assess whether groups were similar by comparing the relative strength of two competing models^25^. We calculated the approximate Bayes factor (BF) with the ‘generalTestBF’ function of the ‘BayesFactor’ R package.

### Experiment 2: Individual differences of quantity discrimination abilities

#### Subjects and apparatus

We tested 36 adult male and female fish (18 of each sex). For this experiment, we used three types of tanks.

**Figure 3:**
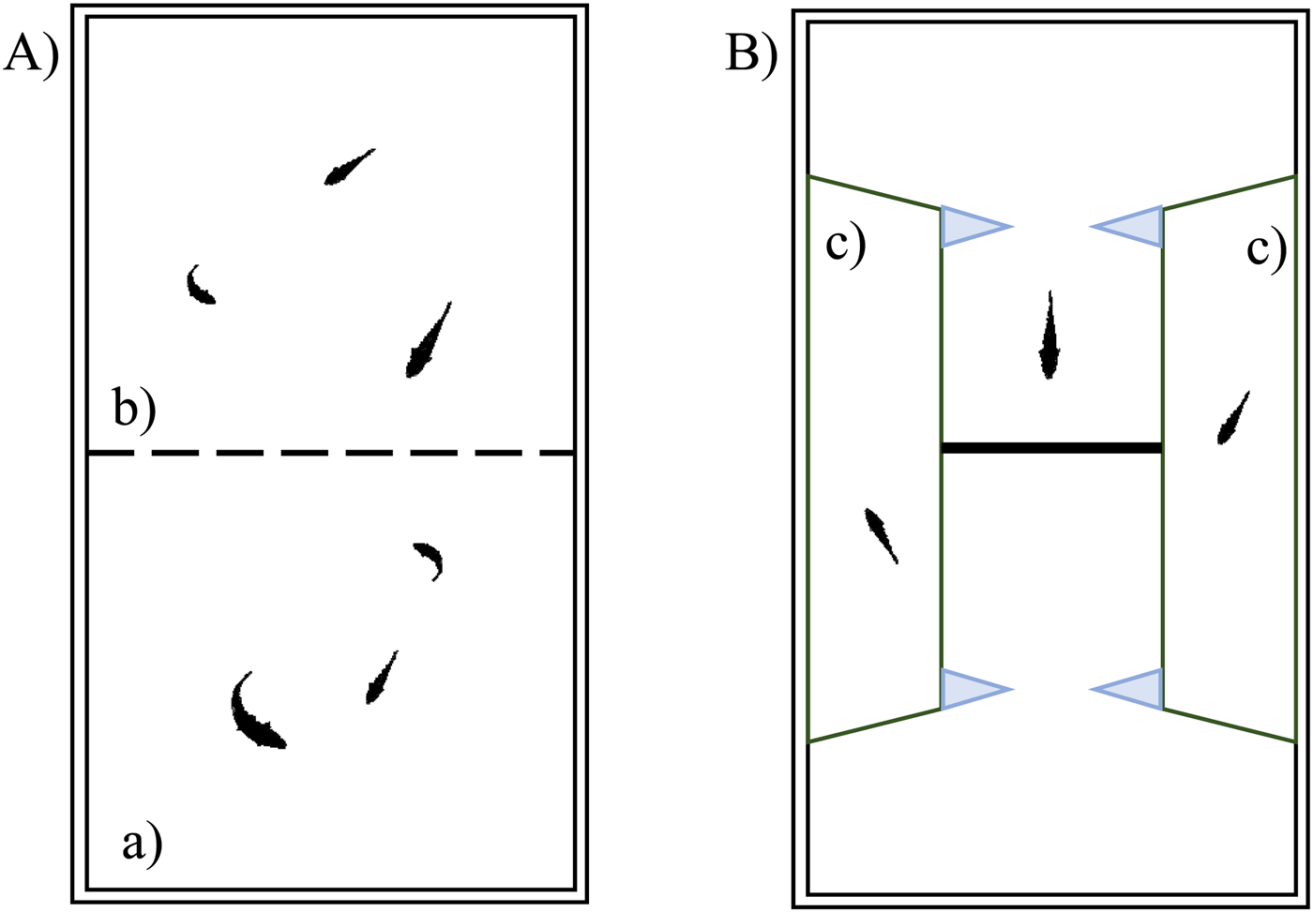
Housing and experimental tank of Experiment 2. A male and a female were housed in the two identical compartments (a and b) of the housing tank (**A**). The experimental tank (**B**) was a scaled-down version of the tank of Experiment 1 with an immature conspecific in each lateral compartment (c).

Housing tanks. To allow individual recognition subjects were maintained in pairs (one male and one female) in specific housing tanks. Each tank (50 × 20 × 32 cm) was provided with natural gravel bottom, plants and a biomechanical filter and was subdivided in two equal compartments by means of a pierced plastic sheet that allowed visual and olfactive communication. The male and the female were housed in each compartment with two immature conspecifics each as social companions (Fig. 3A). Fish were fed with commercial food flakes (Aqua tropical, Padovan®) in the morning, and live brine shrimps, *Artemia salina*, in the afternoon. We arranged nine identical housing tanks in the same dark room. Water temperature was maintained at 26 °C and one 30 W fluorescent lamp illuminated each compartment according to a 12 h:12 h light/dark photoperiod.

Pre-test tank. Prior to the individual test, subjects were allowed to familiarize in group to move between compartments and pass through holes. We used the same apparatus described in experiment 1, provided with a single large hole (2.7 cm in diameter) in the middle of the panel.

Experimental tank. The experiment was conducted in a 50 × 20 × 32 cm glass tank filled with 25 cm of water (Fig. 3B). The experimental apparatus was a scaled-down version of the tank of experiment 1 with two differences: no plant was inserted in the tank and the two immature conspecifics were inserted in the lateral compartments (Fig. 3B). We used two identical apparatuses at the same time, both in the same room of the housing tanks.

#### Procedure and stimuli

Four days before the test, three naïve males and three naïve females were randomly selected and moved to three housing tanks. Since the whole experiment lasted more than three weeks, to avoid stress from social isolation, two fish, one male and one female were housed in the same tank. After two days of habituation in pairs to their housing tank, all six fish were moved together to the pre-test tank. They were maintained in such tank for two days during which they could habituate to swim through the hole; at the end of the second day they were moved again to the housing tanks and a unique ID was assigned to each fish. On the test days, the experimental apparatuses were filled with half new water and half water from the 400-litre tanks in which the zebrafish populations were maintained. One immature zebrafish was inserted in each lateral compartment. After 30 minutes, a subject was inserted in the experimental tank and after 5 minutes the test began. Subjects were presented with three out of the four ratios of experiment 1: ratio 0.60, ratio 0.75 and ratio 0.86. We used the same panels of experiment 1. The presentation order of the ratios was maintained identical for all subjects, from the easiest discrimination to the most difficult one. The first ratio presented was the ratio 0.60; after 30 minutes the panel was flipped to change the relative position of the two holes. After 30 minutes the panel was substituted with the panel of ratio 0.75 and the procedure was repeated until each subject was tested for a total of three consecutive hours (one hour for each ratio). After a subject finished the test, it was moved to its housing tank. The test was repeated for each subject three times, at a week interval.

#### Data collection and statistical analyses

From the video recordings, we scored the number of passages through each hole for every panel presented. One-third of the videos of each subject were analyzed by two different experimenters to assess interrater reliability. Analyses were performed in R version 3.4.2. We used binomial tests to compare the passages through the bigger hole in all three ratios with chance level for both males and females. A linear mixed-effects model (LMM, ‘lmer’ function of the ‘lme4’ R package) was performed to compare the total number of passages between the four ratios. To assess the effect of replication, sex and ratio, we used a LMM fitted with subject ID as random effect. Subsequent pairwise comparisons were performed with Tukey post-hoc tests. To assess differences between subjects, we performed a test of the random-effect term of the LMM (‘ranova’ function of the ‘lmerTest’ R package). The reliability of the test was estimated with Pearson’s correlation coefficients across the three replications, and with Intraclass Coefficients (ICC, ‘icc’ function of the ‘lme4’ R package). A high ICC value reflects high similarity between performances. We calculated the approximate Bayes factor (BF) with the ‘generalTestBF’ function of the ‘BayesFactor’ R package in the case of a lack of significant effect of the sex and of the replication. Binomial tests were performed to compare the passages through the bigger hole in every ratio with chance level for each subject. We performed the Tau-U statistic to test for data overlap between the three repetitions^26^. Smaller differences in individual performance between repetitions reflect higher overlap. We compared the individual performance between two repetitions at once within each subject, for all three ratios; subsequently, we calculated an aggregate value weighted by the number of repetitions^26^.

### Experiment 3: The effect of experience on discrimination abilities

#### Subjects, apparatus, procedure and stimuli

Subjects were 18 females that participated in Experiment 1 and have been maintained in group until the beginning of this experiment and then individually tested as in Experiment 2. Testing apparatus and procedure were the same as Experiment 2 but in this experiment, fish were tested only once after two days in the pre-test tank. This experiment was run at the same time as Experiment 2.

#### Data collection and statistical analyses

All analyses were performed in R version 3.4.2. One-third of the videos of each female were analyzed by two different experimenters to assess interrater reliability. We compared the performances of the 18 females and of the first replication of the 18 females that participated in experiment 2 performing a LMM fitted with experience and ratio as fixed effect and subject ID as a random effect. Binomial tests were performed to compare the passages through the bigger hole in all three ratios with chance level for each female. We calculated the approximate Bayes factor (BF) with the ‘generalTestBF’ function of the ‘BayesFactor’ R package in the case of a lack of significant effect of the experience.

### Ethical notes

The experiments adhered to the current legislation of the country in which they were performed (Italy, Decreto Legislativo 4 Marzo 2014, n. 26) and were approved by the Ethical Committee of the Università di Padova (protocol n. 61/2018).

## Results

### Experiment 1: Group quantity discrimination abilities

The analysis of the number of passages was highly reliable between the two experimenters (Pearson correlation coefficient: *r* = 0.975, *P* < 0.001). The mean number of passages was 2313 ± 586 in 36 h of recordings, namely 386 per hour. The total number of passages did not significantly differ among the four ratios (LMM: *F*_(3, 15)_ = 2.881, *P* = 0.071, Table 3).

**Table 3:** Performance of the six groups tested in Experiment 1.

Group	Ratio 0.60	Ratio 0.75	Ratio 0.86	Ratio 0.91
Group 1	1151/1640 *P* < 0.05*	1098/1833 *P* < 0.05*	961/1765 *P* < 0.05*	1160/1973 *P* < 0.05*
Group 2	1194/1691 *P* < 0.05*	1323/2161 *P* < 0.05*	1075/2028 *P* < 0.01*	1158/2025 *P* < 0.05*
Group 3	2165/3153 *P* < 0.05*	1795/3314 *P* < 0.05*	1568/3070 *P* = 0.241	1570/2855 *P* < 0.05*
Group 4	1585/2205 *P* < 0.05*	1422/2246 *P* < 0.05*	1214/2542 *P* < 0.05*	1326/2532 *P* < 0.05*
Group 5	1784/2432 *P* < 0.05*	1984/3199 *P* < 0.05*	1357/2439 *P* < 0.05*	1980/3599 *P* < 0.05*
Group 6	1145/1552 *P* < 0.05*	1215/1935 *P* < 0.05*	854/1494 *P* < 0.05*	1026/1856 *P* < 0.05*
Mean of the 6 groups	9024/12673 *P* < 0.05*	8837/14688 *P* < 0.05*	7029/13338 *P* < 0.05*	8220/14840 *P* < 0.05*

**Figure 4:**
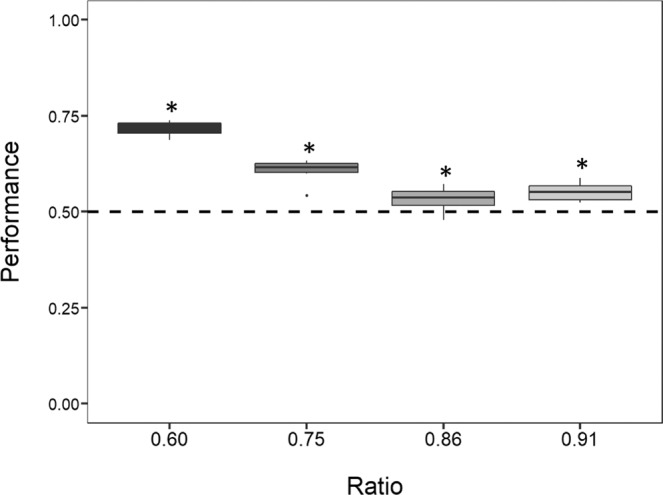
Results of Experiment 1. The Y-axis refers to the proportion of choices for the bigger hole in the four ratios tested. Zebrafish showed to prefer the bigger hole in all four ratios. Asterisks (*) denote a significant departure from chance level (0.5). Bars represent the standard error.

Zebrafish swam significantly more through the bigger hole in all the ratios presented (Fig. 4): ratio 0.60 (mean: 0.712, 95% CI [0.704, 0.720], *P* < 0.001), ratio 0.75 (mean: 0.602, 95% CI [0.594, 0.610], *P* < 0.001), ratio 0.86 (mean: 0.527, 95% CI [0.518, 0.535], *P* < 0.001) and ratio 0.91 (mean: 0.554, 95% CI [0.546, 0.562], *P* < 0.001). The results remained significant after Bonferroni correction for multiple comparisons. There was a significant difference in performances between the four ratios (LMM: *F*_(3, 120)_ = 37.060, *P* < 0.001). A Tukey post hoc test revealed that, with the exception of the pairwise comparison between the ratio 0.86 and the ratio 0.91 (*P* = 0.689), all the other comparisons were statistically significant (all *P* values < 0.05). The effect of the day was not significant (LMM: *F*_(5, 120)_ = 0.753, *P* = 0.586); the interaction between the ratio and the day was also not significant (LMM: *F*_(15, 120)_ = 0.709, *P* = 0.772). The test of the random-effect term of the LMM revealed that the groups did not statistically differ (*P* = 1.000).

#### Control Experiment A: the effect of the size of the habituation hole

**Figure 5:**
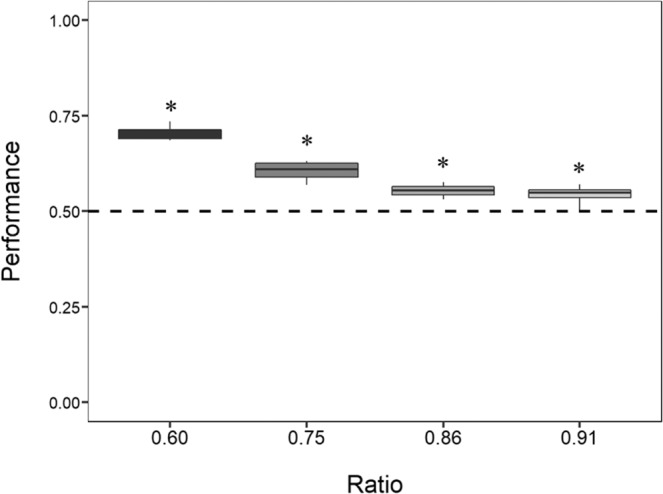
Results of Control Experiment A. The Y-axis refers to the proportion of choices for the bigger hole in the four ratios tested. Zebrafish showed to prefer the larger hole in all four ratios. Asterisks (*) denote a significant departure from chance level (0.5). Bars represent the standard error.

As in Experiment 1, zebrafish swam significantly more through the bigger hole in all the four ratios presented (Table 4, Fig. 5): ratio 0.60 (mean: 0.702, 95% CI [0.694, 0.711], *P* < 0.001), ratio 0.75 (mean: 0.602, 95% CI [0.594, 0.611], *P* < 0.001), ratio 0.86 (mean: 0.556, 95% CI [0.548, 0.564], *P* < 0.001) and ratio 0.91 (mean: 0.545, 95% CI [0.536, 0.553], *P* < 0.001). The results remained significant after Bonferroni correction for multiple comparisons. A significant difference in performances between the four ratios (LMM: *F*_(3, 72)_ = 31.051, *P* < 0.001) was observed, as in Experiment 1. A Tukey post hoc test revealed that, with the exception of the pairwise comparison between the ratio 0.86 and the ratio 0.91 (*P* = 0.960), all the other comparisons were statistically significant (all *P* values < 0.05). The effect of the day was not significant (LMM: *F*_(5, 72)_ = 0.390, *P* = 0.854); the interaction between the ratio and the day was also not significant (LMM: *F*_(15, 72)_ = 0.611, *P* = 0.856). When a LMM was conducted on the pooled data of Experiment 1 and Control Experiment A, adding the diameter of the habituation hole as a factor, no significant difference in performances was found between the two treatments (LMM: *F*_(1, 192)_ = 0.072, *P* = 0.788). The approximate Bayes factor (BF = 0.14) indicated that the LMM model with no effect of the habituation hole diameter is 88 times more likely to explain the performance of the subjects than the model with that effect. The effect of the habituation hole could be greater at the beginning of the test phase. The analysis restricted to the first 3 test hours of test, reached the same conclusion *F*_(1, 32)_ = 0.735, *P* = 0.398).

**Table 4:** Performance of the four groups tested in Control Experiment A.

Group	Ratio 0.60	Ratio 0.75	Ratio 0.86	Ratio 0.91
Group 1	2142/3153 *P* < 0.05*	2132/3735 *P* < 0.05*	2394/4216 *P* < 0.05*	1803/3390 *P* < 0.05*
Group 2	1328/1777 *P* < 0.05*	1300/2072 *P* < 0.05*	1264/2272 *P* < 0.01*	1312/2365 *P* < 0.05*
Group 3	1960/2762 *P* < 0.05*	1717/2698 *P* < 0.05*	1358/2402 *P* = 0.241	1133/2057 *P* < 0.05*
Group 4	2459/3538 *P* < 0.05*	2198/3692 *P* < 0.05*	2618/4842 *P* < 0.05*	3104/5682 *P* < 0.05 *
Mean of the 4 groups	7889/11230 *P* < 0.05*	7347/12197 *P* < 0.05*	7634/13732 *P* < 0.05*	7352/13494 *P* < 0.05*

#### Control Experiment B: the effect of the absolute size of the test holes

**Figure 6:**
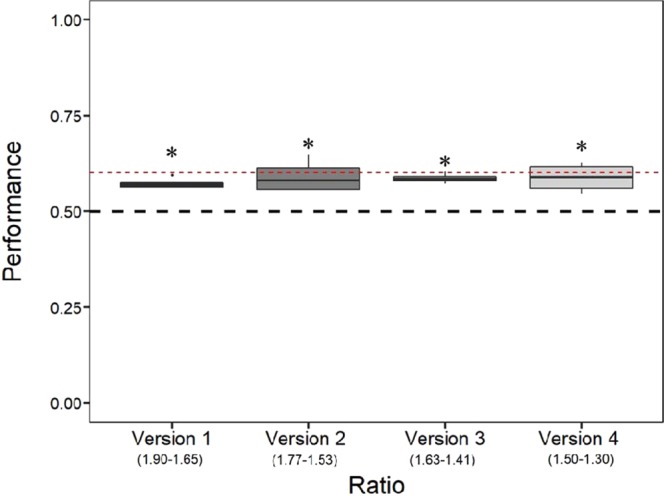
Results of Control Experiment B. The Y-axis refers to the proportion of choices for the bigger hole in the four versions (ratio 0.75) tested. Values in brackets represents the diameters of the larger and the smaller hole. Asterisks (*) denote a significant departure from chance level (0.5). Bars represent the standard error. The dashed red line represents the zebrafish’ performance in the ratio 0.75 in the Experiment 1 (mean = 0.602).

Zebrafish swam significantly more often through the bigger hole in all the four versions of the ratio 0.75 (Table 5, Fig. 6): Version 1 (mean: 0.571, 95% CI [0.562, 0.581], *P* < 0.001), Version 2 (mean: 0.584, 95% CI [0.573, 0.595], *P* < 0.001), Version 3 (mean: 0.577, 95% CI [0.567, 0.588], *P* < 0.001) and Version 4 (mean: 0.578, 95% CI [0.567, 0.589], *P* < 0.001). The results remained significant after Bonferroni correction for multiple comparisons. The LMM revealed no significant difference between the performances in the four versions (*F*_(3, 69)_ = 0.798, *P* = 0.499) and no effect of the day (*F*_(5, 69)_ = 0.460, *P* = 0.804). Also, the interaction between the version and the day was not significant (LMM: *F*_(15, 70)_ = 1.111, *P* = 0.363). When results were compared with the zebrafish’ performance in ratio 0.75 in Experiment 1, the LMM revealed no significant difference (*F*_(4, 24)_ = 0.616, *P* = 0.655). The approximate Bayes factor (BF = 0.10) indicated that the LMM model with no difference between the performances in the ratio 0.75 of the Experiment 1 and the four versions of the Control Experiment B is 91 times more likely to explain the performance of the subjects than the model that includes that difference.

**Table 5:** Performance of the four groups tested in Control Experiment B.

Group	Ratio 0.60	Ratio 0.75	Ratio 0.86	Ratio 0.91
Group 1	2183/3837 *P* < 0.05*	1681/3017 *P* < 0.05*	1497/2641 *P* < 0.05*	1369/2533 *P* < 0.05*
Group 2	1210/2039 *P* < 0.05*	1139/1784 *P* < 0.05*	1078/1847 *P* < 0.01*	894/1433 *P* < 0.05*
Group 3	1382/2470 *P* < 0.05*	1097/1964 *P* < 0.05*	1371/2366 *P* = 0.241	993/1759 *P* < 0.05*
Group 4	1130/1992 *P* < 0.05*	693/1589 *P* < 0.05*	795/1115 *P* < 0.05*	1094/1799 *P* < 0.05*
Mean of the 4 groups	5905/10338 *P* < 0.05*	4880/8354 *P* < 0.05 *	5061/8764 *P* < 0.05*	4350/7524 *P* < 0.05*

### Experiment 2: Individual differences of quantity discrimination abilities

The analysis of the number of passages was highly reliable between the two experimenters (Pearson correlation coefficient: *r* = 0.995, *P* < 0.001). The mean total number of passages in ratio 0.60 was 88 ± 41 (mean ± SD) for the 18 males and 84 ± 55 for the 18 females. The mean total number of passages in ratio 0.75 was 108 ± 50 for the males and 107 ± 71 for the females. The mean total number of passages in ratio 0.86 was 105 ± 53 for the 18 males and 114 ± 71 for the 18 females.

**Figure 7:**
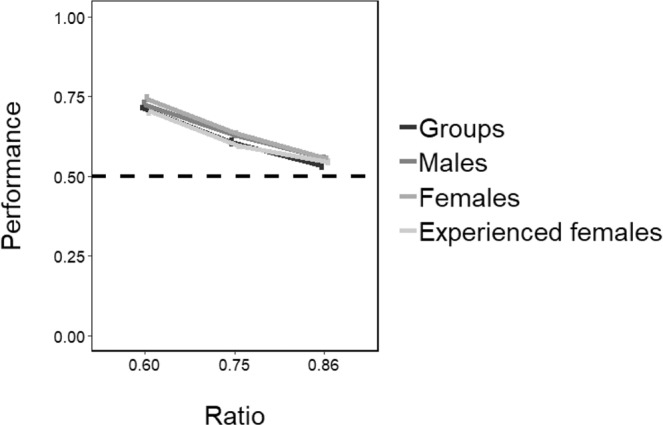
Comparison of the results of Experiment 1 and 2. The Y-axis refers to the proportion of choices for the bigger hole in the three ratios tested (ratio 0.60, ratio 0.75, ratio 0.86). Bars represent the standard error.

Overall, males performed a mean proportion of 0.726 ± 0.051 (mean ± SD; 95% CI [0.713, 0.738], *P* < 0.001) passages through the bigger hole in ratio 0.60, 0.692 ± 0.06 (95% CI [0.680, 0.704], *P* < 0.001) in ratio 0.75 and 0.569 ± 0.039 (95% CI [0.556, 0.582], *P* < 0.001; Fig. 7) in ratio 0.86. Females performed a mean proportion of 0.724 ± 0.04 (95% CI [0.704, 0.728], *P* < 0.001) passages through the bigger hole in ratio 0.60, 0.634 ± 0.054 (95% CI [0.621, 0.647], *P* < 0.001) in ratio 0.75 and 0.532 ± 0.036 (95% CI [0.520, 0.544], *P* < 0.001; Fig. 7) in ratio 0.86. The results remained significant after Bonferroni correction for multiple comparisons.

The LMM revealed a significant effect of the ratio (*F*_(2, 272)_ = 310.354, *P* < 0.001) on the subjects’ performance. A Tukey post hoc test revealed that all pairwise comparisons between the ratios were statistically significant (all *P*-values < 0.001). There was no significant effect of sex (LMM: *F*_(1, 34)_ = 0.001, *P* = 0.981) and no effect of the replication (*F*_(2, 272)_ = 0.640, *P* = 0.528). All interactions were not significant (all *P*-values > 0.528). The test of the random-effect term of the LMM revealed that the subjects statistically differed (*P* < 0.001). The approximate Bayes factor (BF = 0.24) indicated that the LMM model with no difference between males and females is 81 times more likely to explain the performance of the subjects than the model that includes such difference. The approximate Bayes factor (BF = 0.12) indicated that the LMM model with no differences between the replications is 89 times more likely to explain the performance of the subjects than the model that includes those differences.

Individual binomial tests revealed that 17 males out of 18 and 17 females out of 18 demonstrated to significantly discriminate the bigger hole in ratio 0.60; 10 males and 13 females significantly discriminated the bigger hole in ratio 0.75 while only 2 males and 4 females discriminated it in the most difficult ratio, ratio 0.86. Figure 6 represents the proportion of choices for the bigger hole in the three ratios tested for each subject. Test-retest reliability coefficients (Pearson’s correlation coefficients and Intraclass Coefficients) revealed that the two most informative ratios are the easiest ones (ratio 0.60 and ratio 0.75) and that only the intermediate ratio gave a good result in terms of reliability (Table 6). The individual TAU-U statistic revealed that the performance did not significantly differ between the three repetitions within each of the 18 males and 18 females. Binomial tests on the individual performances in every ratio for each replication and all TAU-U statistics are available in the supplementary materials.

**Table 6:** Performance of zebrafish individually tested in Experiment 2. Descriptive Statistics (Mean ± SD), Pearson’s Correlation Coefficients (r) and Intraclass Coefficients (ICC) for the three ratios and for the combination of ratios. Significant results of the Pearson’s correlation are in bold. When both coefficients (r and ICC) are close to 1, it indicates high similarity between the performances.

	Replication 1	Replication 2	Replication 3	r_1-2_	r_1-3_	r_2-3_	ICC
	Mean ± SD	Mean ± SD	Mean ± SD				
Ratio 0.60	0.732 ± 0.064	0.736 ± 0.075	0.739 ± 0.063	**0.375**	0.285	**0.728**	0.474
Ratio 0.75	0.645 ± 0.064	0.631 ± 0.055	0.625 ± 0.055	**0.523**	**0.476**	**0.553**	0.500
Ratio 0.86	0.560 ± 0.056	0.545 ± 0.060	0.555 ± 0.046	0.065	**0.552**	**0.440**	0.323
Mean ratios 0.60–0.75	0.688 ± 0.053	0.683 ± 0.052	0.682 ± 0.046	**0.584**	**0.421**	**0.695**	0.570
Mean ratios 0.75–0.86	0.602 ± 0.052	0.588 ± 0.042	0.590 ± 0.039	0.193	**0.592**	**0.512**	0.401
Mean ratios 0.60–0.75–0.86	0.645 ± 0.047	0.637 ± 0.041	0.640 ± 0.035	**0.358**	**0.665**	**0.466**	0.475

### Experiment 3: The effect of experience on discrimination abilities

**Figure 8:**
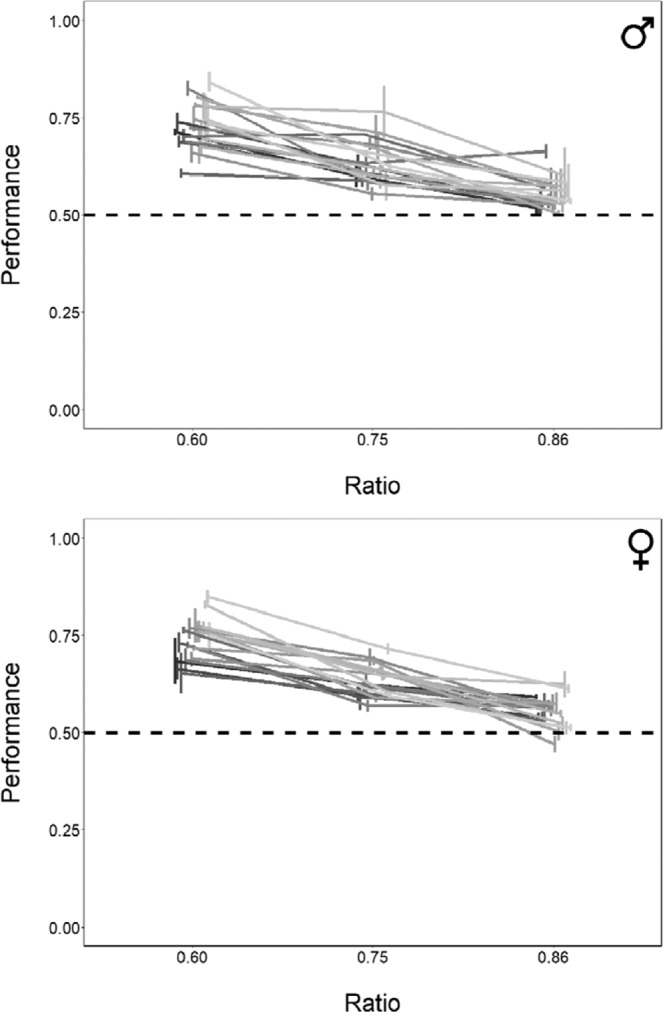
Results of Experiment 2. The Y-axis refers to the mean proportion of choices for the bigger hole in the three ratios tested for each subject (males: upper graph, females: lower graph). Bars represent the standard error.

The analysis of the number of passages was highly reliable between the two experimenters (Pearson correlation coefficient: *r* = 0.989, *P* < 0.001). The mean total number of passages in ratio 0.60 was 61 ± 42, in ratio 0.75 was 88 ± 42 and in ratio 0.86 was 78 ± 46 for the 18 experienced females. Overall, experienced females performed a mean proportion of 0.706 ± 0.053 (mean ± SD; 95% CI [0.676, 0.736], *P* < 0.001) passages through the bigger hole in ratio 0.60, 0.595 ± 0.077 (95% CI [0.569, 0.618], *P* < 0.001) in ratio 0.75 and 0.543 ± 0.049 (95% CI [0.517, 0.570], *P* < 0.001; Fig. 8) in ratio 0.86. The results remained significant after Bonferroni correction for multiple comparisons. The LMM revealed no significant effect of experience (*F*_(1, 19)_ = 3.417, *P* = 0.080) and no experience x ratio interaction effect was found (*F*_(2, 38)_ = 1.577, *P* = 0.220). Individual binomial tests revealed that 15 experienced females out of 18 significantly discriminated the bigger hole in ratio 0.60, 8 in ratio 0.75 and only one in ratio 0.86. The approximate Bayes factor (BF = 0.54) indicated that the LMM model with no difference between naïve and experienced females is 65 times more likely to explain the performance of the subjects than the model that includes that difference.

## Discussion

In this study we developed an easy method based on spontaneous preference to measure the ability to discriminate continuous quantities in zebrafish. In Experiment 1, we collected normative data to compare individual performances. In particular, we tested six groups of six residential fish for 36 hours during which they could spontaneously move in their tanks passing through one of two holes. The results show that zebrafish have a good ability to discriminate between areas as shown by the preference to pass through the larger hole even in the most difficult discrimination, 0.91 areas ratio. As expected from Weber’s law, discrimination ability decreased as the ratio between the holes increased, ranging from around 72% with 0.60 ratio to around 55% for the 0.91 ratio. Ratios 0.87 and 0.91 were just above 50%, suggesting that they are probably close to the discriminability threshold of zebrafish. In our experiment, it was not assessed if fish were comparing the size of holes using a linear measure, such as the diameter or the perimeter, or they discriminated between areas. However, the hypothesis that they were comparing areas is the most conservative of the two since the ratio between the linear measures is much larger (from 0.77 to 0.96; Table 1).

One may argue that zebrafish could have applied a generalization rule to solve the task and that the preference for passing through the larger hole in the test phase was determined by the use of a single large hole during the habituation phase. However, when in Control Experiment A fish were habituated using a hole smaller than the one used in Experiment 1, we obtained the same results, thus excluding the possibility of a generalization mechanism at the basis of the fish choice.

Another potential weakness of Experiment 1 is that we use a single version for each ratio. As a consequence of the fact that the ratio was always adjusted by varying the size of the smaller hole, as the ratio between holes increased, the average absolute size of the holes also increases. This makes it difficult to sort apart the influence of the ratio and absolute size on size discrimination. To unravel this problem, in Control Experiment B zebrafish were presented with four different versions that greatly varied in absolute size but had the same ratio. The performance did not change with absolute size suggesting that zebrafish were spontaneously comparing the sizes of the two holes, rather than using the absolute size of each hole as reference to take a decision.

Together, these Control Experiments A and B also answer to another possible limitation of Experiment 1. Since we kept constant the size of the larger hole and the ratio was adjusted by varying the size of the smaller one, during testing, subjects had substantially more exposure to the larger hole. Yet, both control experiments strongly indicate that zebrafish respond to the relative size of two holes and are totally unaffected by being exposed to large or small holes before or during the experiment.

Lastly, one may argue that our experiment does not allow distinguishing with absolute certainty between the discrimination and the preference for the larger hole. It is acknowledged that motivation represents a critical aspect in spontaneous choice tests as a lack of preference does not necessarily implies a lack of discrimination. This issue concerns several research fields. For instance, if an animal does not show a preference for picking 11 pieces of food over 10, it may be possible that the absence of choice is due to the fact that both amounts provide enough energy, thus reflecting a functional threshold and not a limit intrinsic to the representational system. This was not the case in our study, as fish successfully chose the larger hole in all the contrasts presented. Yet we cannot exclude the possibility that potential null results simply reflect a change in the individuals’ motivational state rather than a true limit in cognitive abilities. One way to overcome this potential limitation consists in using conditioning procedure where animals are trained to associate stimuli to a reward. Since motivation to get the reward is supposed to be the same irrespective of the stimuli presented, a lack of choice is more likely to reflect the upper threshold of the discriminative abilities of the individuals. However, training procedures are often time-consuming, potentially stressful and their ecological validity is questionable (;^27,28^). Furthermore, it has been argued that extensive training may lead to recruitment of neurocognitive systems that may normally have different functions thus mimicking cognitive abilities that are not in the natural repertoire of the species (;^28,29^). Finally training procedures are unavailable or even impossible to implement for many species and for entire taxa, thus preventing any possibility of investigation in these organisms and considerably limiting cross-species comparison.

A capacity to discriminate areas that differ for less than 10% indicates high discrimination abilities in zebrafish. By comparison, adult humans are only slightly better, as they can discriminate between areas up to a 0.95 ratio^30^. Few other species have been tested in similar tasks. Two California sea lions tested underwater, were able to discriminate geometrical shapes, one up to a 0.88 size ratio and the other up to a 0.94 ratio^31^. Guppies accurately discriminate two pieces of food of different size. Accuracy ranges from 90% for a 0.25 ratio to approximately 65% for 0.75 ratio. It is unknown whether they can achieve more difficult discriminations^22^. As far as we are aware, this is the very first evidence of such a precise quantitative discrimination in a fish species (^16,17^).

Previously, some studies investigated numerical discrimination ability in zebrafish. Zebrafish were found to discriminate 8 from 12 items (ratio 0.67) but not 9 from 12 items (ratio 0.75)^18^. In another study, zebrafish were able to discriminate 1 vs. 2 conspecifics (ratio 0.50) and 2 vs. 3 conspecifics (ratio 0.67) but failed to discriminate a more difficult comparison, 3 vs. 4 conspecifics (ratio 0.75)^19^. Therefore, it seems that this species is more accurate in discriminating between areas than between numerosities.

In the first experiment, we studied fish in social groups, the same condition in which this highly social species is expected to solve this type of problem in nature. This condition also allowed us to conduct long-lasting experiments without the risk that isolation could cause stress and induce pathological modifications of behaviour. However, in biomedical and genetic research it is generally necessary to test subjects individually. Therefore, in the second experiment we tested individual subjects in the same test but for shorter observation times. On average, individual’s performance overlap that of fish tested in groups but a large individual variation in performance was observed. When the same subjects were tested again after 7 and 14 days, they tended to repeat the same performance and no effect of repetition was observed. The two ratios that better differentiate individuals are the easiest ones, ratio 0.67 and ratio 0.75, and in particular this latest one. Therefore, future studies could be performed only with this ratio. This experiment tends to exclude an effect of experience on performance. Nonetheless, in this experiment subjects accumulated an experience from a previous trial of only 3 hours, a time that may not be enough to influence the performance. In Experiment 3, we re-tested 18 females that participated in Experiment 1 and compared their scores with those of unexperienced females (the first replication of the females of Experiment 2). Even in this case, we did not find any effect of practice, despite these females having had a total of 36 hours of testing experience. This lack of an effect could be of fundamental importance for those studies that investigate cognitive decline in normal and pathological aging or test drug effects on diseases that cause cognitive deficits.

## Conclusion

Zebrafish is increasingly used as a model in biomedical research, in particular for translational research on human neuropathologies and aging. There have been impressive progresses in development of molecular tools to study these aspects. By converse much less progress have been made in developing tools that allow assessment of the cognitive changes in performance that regularly accompany many of these pathologies. Our study gives a small contribution towards the development of a battery of measurements and tests that can be used for this purpose.

## Supplementary information

Individual performances and statistics.
