# Supplementary material for: Size discrimination in adult zebrafish (Danio rerio): Normative data and individual variation
Source: Sci Rep. 2020 Jan 24;10:1164. doi: 10.1038/s41598-020-57813-1 (PMC6981261; doi:10.1038/s41598-020-57813-1)
Supplement: Supplementary file 1 — Individual performances and statistics. [file 41598_2020_57813_MOESM1_ESM.docx]

**Size discrimination in adult zebrafish (*Danio rerio*): Normative data and individual variation**

Maria Santacà^1^*, Tiziano Caja^2^, Maria Elena Miletto Petrazzini^3^, Christian Agrillo^1^ & Angelo Bisazza^1,4^

^1^ Department of General Psychology, University of Padova, Padova, Italy

^2^ Department of Life Sciences and Systems Biology, University of Torino, Torino, Italy

^3^ School of Biological and Chemical Sciences, Queen Mary University of London, UK

^4^ Padova Neuroscience Center, University of Padova, Padova, Italy

*Corresponding author: Maria Santacà. Department of General Psychology, Via Venezia 8, 35131 Padova (Italy). Phone: +39 0498277424, e-mail: [santacamaria@gmail.com](mailto:santacamaria@gmail.com)

**Binomial tests and TAU-U statistics on the individual performances in every ratio for each replication.**

| Subject ID | Sex | Replication | Ratio 0.60 | Ratio 0.75 | Ratio 0.86 | Tau-U statistic |
| --- | --- | --- | --- | --- | --- | --- |
| 1M | M | 1 | 35/51  *P* < 0.05 * | 46/77  *P* = 0.110 | 39/78  *P* = 1.000 | Tau = 0.037  *P* = 0.900 |
| 1M | M | 2 | 60/79  *P* < 0.05 * | 52/87  *P* = 0.086 | 59/101  *P* = 0.111 |  |
| 1M | M | 3 | 79/99  *P* < 0.05 * | 44/76  *P* = 0.207 | 27/51  *P* = 0.780 |  |
| 2M | M | 1 | 75/94  *P* < 0.05 * | 89/122  *P* < 0.05 * | 25/41  *P* = 0.211 | Tau = -0.185  *P* = 0.529 |
| 2M | M | 2 | 82/104  *P* < 0.05 * | 109/144  *P* < 0.05 * | 42/82  *P* = 0.912 |  |
| 2M | M | 3 | 91/118  *P* < 0.05 * | 133/212  *P* < 0.05 * | 106/186  *P* = 0.067 |  |
| 3M | M | 1 | 81/101  *P* < 0.05 * | 83/112  *P* < 0.05 * | 42/84  *P* = 1.000 | Tau = -0.185  *P* = 0.529 |
| 3M | M | 2 | 103/127  *P* < 0.05 * | 109/165  *P* < 0.05 * | 86/166  *P* = 0.698 |  |
| 3M | M | 3 | 61/77  *P* < 0.05 * | 90/148  *P* < 0.05 * | 117/238  *P* = 0.846 |  |
| 4M | M | 1 | 164/218  *P* < 0.05 * | 78/128  *P* < 0.05 * | 49/76  *P* < 0.05 * | Tau = -0.111  *P* = 0.706 |
| 4M | M | 2 | 102/172  *P* < 0.05 * | 118/200  *P* < 0.05 * | 98/157  *P* < 0.05 * |  |
| 4M | M | 3 | 65/95  *P* < 0.05 * | 110/170  *P* < 0.05 * | 70/120  *P* = 0.082 |  |
| 5M | M | 1 | 66/108  *P* < 0.05 * | 76/120  *P* < 0.05 * | 63/128  *P =* 0.930 | Tau = 0.185  *P* = 0.529 |
| 5M | M | 2 | 71/100  *P* < 0.05 * | 70/116  *P* < 0.05 * | 78/137  *P =* 0.124 |  |
| 5M | M | 3 | 47/61  *P* < 0.05 * | 47/76  *P* < 0.05 * | 44/84  *P =* 0.744 |  |
| 6M | M | 1 | 57/69  *P* < 0.05 * | 26/29  *P* < 0.05 * | 35/48  *P* < 0.05 * | Tau = -0.556  *P* = 0.059 |
| 6M | M | 2 | 20/25  *P* < 0.05 * | 23/32  *P* < 0.05 * | 10/21  *P =* 1.000 |  |
| 6M | M | 3 | 72/101  *P* < 0.05 * | 66/93  *P* < 0.05 * | 50/84  *P =* 0.101 |  |
| 7M | M | 1 | 60/90  *P* < 0.05 * | 22/35  *P =* 0.176 | 36/61  *P =* 0.200 | Tau = -0.111  *P* = 0.706 |
| 7M | M | 2 | 20/23  *P* < 0.05 * | 25/50  *P* = 1.000 | 103/181  *P =* 0.074 |  |
| 7M | M | 3 | 100/142  *P* < 0.05 * | 76/127  *P* < 0.05 * | 46/87  *P =* 0.668 |  |

| 8M | M | 1 | 56/75  *P* < 0.05 * | 21/41  *P =* 1.000 | 46/78  *P =* 0.668 | Tau = 0.259  *P* = 0.378 |
| --- | --- | --- | --- | --- | --- | --- |
| 8M | M | 2 | 47/71  *P* < 0.05 * | 77/116  *P =* 0.145 | 35/56  *P =* 0.081 |  |
| 8M | M | 3 | 74/88  *P* < 0.05 * | 52/75  *P* < 0.05 * | 51/102  *P =* 1.000 |  |

| 9M | M | 1 | 84/97  *P* < 0.05 * | 101/170  *P* < 0.05 * | 72/138  *P* = 0.671 | Tau = 0.111  *P* = 0.706 |
| --- | --- | --- | --- | --- | --- | --- |
| 9M | M | 2 | 40/49  *P* < 0.05 * | 49/77  *P* < 0.05 * | 45/84  *P* = 0.586 |  |
| 9M | M | 3 | 20/25  *P* < 0.05 * | 28/42  *P* < 0.05 * | 34/62  *P* = 0.526 |  |
| 10M | M | 1 | 69/95  *P* < 0.05 * | 90/148  *P* < 0.05 * | 90/184  *P* = 0.825 | Tau = -0.185  *P* = 0.529 |
| 10M | M | 2 | 100/139  *P* < 0.05 * | 74/114  *P* < 0.05 * | 90/162  *P* = 0.182 |  |
| 10M | M | 3 | 83/119  *P* < 0.05 * | 85/156  *P* = 0.298 | 83/162  *P* = 0.814 |  |
| 11M | M | 1 | 48/61  *P* < 0.05 * | 38/65  *P* = 0.215 | 25/53  *P* = 0.784 | Tau = 0.074  *P* = 0.801 |
| 11M | M | 2 | 83/116  *P* < 0.05 * | 78/119  *P* < 0.05 * | 65/119  *P* = 0.359 |  |
| 11M | M | 3 | 55/65  *P* < 0.05 * | 24/29  *P* < 0.05 * | 41/79  *P* = 0.820 |  |
| 12M | M | 1 | 28/42  *P* < 0.05 * | 36/60  *P* = 0.155 | 46/92  *P* = 1.000 | Tau = 0.185  *P* = 0.529 |
| 12M | M | 2 | 40/59  *P* < 0.05 * | 52/85  *P* = 0.051 | 48/95  *P* = 1.000 |  |
| 12M | M | 3 | 41/57  *P* < 0.05 * | 50/87  *P* = 0.198 | 103/180  *P* = 0.062 |  |
| 13M | M | 1 | 43/70  *P* = 0.072 | 65/110  *P* = 0.070 | 52/97  *P* = 0.543 | Tau = -0.259  *P* = 0.379 |
| 13M | M | 2 | 35/59  *P* = 0.193 | 59/99  *P* = 0.070 | 45/89  *P* = 1.000 |  |
| 13M | M | 3 | 43/72  *P =* 0.125 | 61/110  *P* = 0.294 | 79/140  *P* = 0.151 |  |
| 14M | M | 1 | 89/146  *P* < 0.05 * | 123/175  *P* < 0.05 * | 78/138  *P* = 0.148 | Tau = 0.407  *P* = 0.166 |
| 14M | M | 2 | 62/99  *P* < 0.05 * | 120/170  *P* < 0.05 * | 11/19  *P* = 0.648 |  |
| 14M | M | 3 | 56/76  *P* < 0.05 * | 90/126  *P* < 0.05 * | 41/72  *P* = 0.289 |  |

| 15M | M | 1 | 16/22  *P* = 0.052 | 26/38  *P* < 0.05 * | 37/59  *P* = 0.067 | Tau = 0.074  *P* = 0.801 |
| --- | --- | --- | --- | --- | --- | --- |
| 15M | M | 2 | 19/23  *P* < 0.05 * | 22/35  *P* = 0.176 | 24/37  *P* = 0.099 |  |
| 15M | M | 3 | 27/41  *P* = 0.059 | 23/39  *P* = 0.338 | 11/16  *P* = 0.210 |  |
| 16M | M | 1 | 110/174  *P* < 0.05 * | 122/204  *P* < 0.05 * | 134/218  *P* < 0.05 * | Tau = -0.111  *P* = 0.706 |
| 16M | M | 2 | 49/57  *P* < 0.05 * | 83/135  *P* < 0.05 * | 71/125  *P* = 0.152 |  |
| 16M | M | 3 | 131/158  *P* < 0.05 * | 86/170  *P* = 0.939 | 117/216  *P* = 0.247 |  |
| 17M | M | 1 | 82/123  *P* < 0.05 * | 114/174  *P* < 0.05 * | 93/145  *P* < 0.05 * | Tau = 0.074  *P* = 0.801 |
| 17M | M | 2 | 76/92  *P* < 0.05 * | 91/124  *P* < 0.05 * | 25/50  *P* = 1.000 |  |
| 17M | M | 3 | 65/80  *P* < 0.05 * | 82/115  *P* < 0.05 * | 82/137  *P* < 0.05 * |  |
| 18M | M | 1 | 63/100  *P* < 0.05 * | 53/93  *P* = 0.213 | 64/116  *P =* 0.307 | Tau = 0.111  *P* = 0.706 |
| 18M | M | 2 | 33/50  *P* < 0.05 * | 36/69  *P =* 0.810 | 43/88  *P =* 0.915 |  |
| 18M | M | 3 | 47/66  *P* < 0.05 * | 44/77  *P =* 0.254 | 50/92  *P =* 0.466 |  |
| 1F | F | 1 | 31/44  *P* < 0.05 * | 29/41  *P* < 0.05 * | 27/56  *P =* 0.894 | Tau = 0.037  *P* = 0.900 |
| 1F | F | 2 | 22/30  *P* < 0.05 * | 18/27  *P =* 0.122 | 18/42  *P =* 0.441 |  |
| 1F | F | 3 | 30/42  *P* < 0.05 * | 15/22  *P =* 0.134 | 32/64  *P =* 1.000 |  |
| 2F | F | 1 | 64/93  *P* < 0.05 * | 73/123  *P* < 0.05 * | 28/54  *P =* 0.892 | Tau = 0.185  *P* = 0.529 |
| 2F | F | 2 | 124/152  *P* < 0.05 * | 114/186  *P* < 0.05 * | 96/157  *P* < 0.05 * |  |
| 2F | F | 3 | 193/235  *P* < 0.05 * | 94/161  *P* < 0.05 * | 64/106  *P* < 0.05 * |  |
| 3F | F | 1 | 36/50  *P* < 0.05 * | 32/49  *P* < 0.05 * | 17/32  *P =* 0.860 | Tau = -0.037  *P* = 0.900 |
| 3F | F | 2 | 77/95  *P* < 0.05 * | 72/107  *P* < 0.05 * | 57/125  *P =* 0.371 |  |
| 3F | F | 3 | 127/170  *P* < 0.05 * | 113/175  *P* < 0.05 * | 173/333  *P =* 0.511 |  |
| 4F | F | 1 | 54/76  *P* < 0.05 * | 60/90  *P* < 0.05 * | 104/187  *P =* 0.143 | Tau = 0.037  *P* = 0.900 |
| 4F | F | 2 | 148/194  *P* < 0.05 * | 236/350  *P* < 0.05 * | 193/343  *P* < 0.05 * |  |
| 4F | F | 3 | 233/289  *P* < 0.05 * | 297/471  *P* < 0.05 * | 171/324  *P =* 0.345 |  |

| 5F | F | 1 | 111/143  *P* < 0.05 * | 101/145  *P* < 0.05 * | 107/193  *P =* 0.150 | Tau = -0.259  *P* = 0.378 |
| --- | --- | --- | --- | --- | --- | --- |
| 5F | F | 2 | 69/89  *P* < 0.05 * | 86/141  *P* < 0.05 * | 59/113  *P =* 0.707 |  |
| 5F | F | 3 | 119/155  *P* < 0.05 * | 113/170  *P* < 0.05 * | 37/75  *P =* 1.000 |  |
| 6F | F | 1 | 73/98  *P* < 0.05 * | 110/168  *P* < 0.05 * | 93/162  *P =* 0.070 | Tau = 0.037  *P* = 0.900 |
| 6F | F | 2 | 47/59  *P* < 0.05 * | 75/114  *P* < 0.05 * | 66/96  *P* < 0.05 * |  |
| 6F | F | 3 | 144/187  *P* < 0.05 * | 116/186  *P* < 0.05 * | 90/147  *P* < 0.05 * |  |
| 7F | F | 1 | 85/103  *P* < 0.05 * | 71/118  *P* < 0.05 * | 82/153  *P =* 0.419 | Tau = -0.037  *P* = 0.900 |
| 7F | F | 2 | 57/67  *P* < 0.05 * | 35/56  *P =* 0.081 | 12/25  *P =* 1.000 |  |
| 7F | F | 3 | 34/42  *P* < 0.05 * | 27/45  *P =* 0.233 | 25/48  *P =* 0.885 |  |
| 8F | F | 1 | 15/17  *P* < 0.05 * | 14/18  *P* < 0.05 * | 11/17  *P* = 0.332 | Tau = -0.259  *P* = 0.378 |
| 8F | F | 2 | 24/45  *P* < 0.05 * | 21/30  *P* < 0.05 * | 32/56  *P* = 0.350 |  |
| 8F | F | 3 | 30/49  *P* < 0.05 * | 64/84  *P* < 0.05 * | 62/105  *P* = 0.078 |  |
| 9F | F | 1 | 7/9  *P* = 0.180 | 19/31  *P* = 0.281 | 14/26  *P* = 0.845 | Tau = -0.333  *P* = 0.257 |
| 9F | F | 2 | 8/10  *P* = 0.109 | 19/29  *P* = 0.136 | 15/32  *P* = 0.860 |  |
| 9F | F | 3 | 8/11  *P* = 0.227 | 7/12  *P* = 0.774 | 4/9  *P* = 1.000 |  |
| 10F | F | 1 | 82/102  *P* < 0.05 * | 73/118  *P* < 0.05 * | 65/112  *P* = 0.108 | Tau = 0.111  *P* = 0.705 |
| 10F | F | 2 | 58/94  *P* < 0.05 * | 97/154  *P* < 0.05 * | 105/176  *P* < 0.05 * |  |
| 10F | F | 3 | 60/95  *P* < 0.05 * | 99/158  *P* < 0.05 * | 104/174  *P* < 0.05 * |  |
| 11F | F | 1 | 62/86  *P* < 0.05 * | 56/87  *P* < 0.05 * | 59/105  *P* = 0.241 | Tau = -0.482  *P* = 0.102 |
| 11F | F | 2 | 49/77  *P* < 0.05 * | 52/90  *P* = 0.170 | 71/132  *P* = 0.434 |  |
| 11F | F | 3 | 47/74  *P* < 0.05 * | 46/81  *P* = 0.266 | 73/139  *P* = 0.611 |  |
| 12F | F | 1 | 53/78  *P* < 0.05 * | 86/131  *P* < 0.05 * | 95/166  *P* = 0.074 | Tau = -0.148  *P* = 0.614 |
| 12F | F | 2 | 38/50  *P* < 0.05 * | 38/69  *P* = 0.470 | 43/77  *P* = 0.362 |  |
| 12F | F | 3 | 37/49  *P* < 0.05 * | 39/69  *P* = 0.336 | 43/77  *P* = 0.362 |  |

| 13F | F | 1 | 68/90  *P* < 0.05 * | 74/121  *P* < 0.05 * | 85/168  *P* = 0.939 | Tau = -0.148  *P* = 0.614 |
| --- | --- | --- | --- | --- | --- | --- |
| 13F | F | 2 | 42/70  *P* = 0.120 | 44/69  *P* < 0.05 * | 59/103  *P* = 0.167 |  |
| 13F | F | 3 | 78/130  *P* < 0.05 * | 76/126  *P* < 0.05 * | 74/143  *P* = 0.738 |  |
| 14F | F | 1 | 50/66  *P* < 0.05 * | 75/122  *P* < 0.05 * | 61/102  *P* = 0.059 | Tau = 0.037  *P* = 0.900 |
| 14F | F | 2 | 32/41  *P* < 0.05 * | 38/64  *P* = 0.169 | 35/72  *P* = 0.906 |  |
| 14F | F | 3 | 27/36  *P* < 0.05 * | 74/114  *P* < 0.05 * | 65/107  *P* < 0.05 * |  |
| 15F | F | 1 | 54/82  *P* < 0.05 * | 84/143  *P* < 0.05 * | 68/118  *P* = 0.117 | Tau = 0.333  *P* = 0.257 |
| 15F | F | 2 | 37/57  *P* < 0.05 * | 45/72  *P* < 0.05 * | 68/117  *P* = 0.096 |  |
| 15F | F | 3 | 44/59  *P* < 0.05 * | 36/53  *P* < 0.05 * | 57/101  *P* = 0.232 |  |
| 16F | F | 1 | 49/68  *P* < 0.05 * | 68/119  *P* = 0.142 | 78/141  *P* = 0.238 | Tau = 0.037  *P* = 0.900 |
| 16F | F | 2 | 49/66  *P* < 0.05 * | 79/133  *P* < 0.05 * | 59/106  *P* = 0.285 |  |
| 16F | F | 3 | 66/93  *P* < 0.05 * | 33/61  *P* = 0.609 | 43/72  *P* = 0.125 |  |
| 17F | F | 1 | 40/53  *P* < 0.05 * | 48/74  *P* < 0.05 * | 36/68  *P* = 0.716 | Tau = 0.185  *P* = 0.529 |
| 17F | F | 2 | 46/63  *P* < 0.05 * | 54/76  *P* < 0.05 * | 37/65  *P* = 0.321 |  |
| 17F | F | 3 | 77/94  *P* < 0.05 * | 97/135  *P* < 0.05 * | 71/125  *P* = 0.152 |  |
| 18F | F | 1 | 73/112  *P* < 0.05 * | 53/81  *P* < 0.05 * | 54/87  *P* < 0.05 * | Tau = 0.111  *P* = 0.706 |
| 18F | F | 2 | 28/39  *P* < 0.05 * | 29/45  *P* = 0.072 | 57/93  *P* < 0.05 * |  |
| 18F | F | 3 | 46/66  *P* < 0.05 * | 29/44  *P* < 0.05 * | 54/88  *P* < 0.05 * |  |
| 19F | Experienced F | 1 | 117/195  *P* < 0.05 * | 70/120  *P* = 0.082 | 65/123  *P* = 0.589 | N/A |
| 20F | Experienced F | 1 | 14/24  *P* = 0.541 | 64/106  *P* < 0.05 * | 16/27  *P* = 0.442 | N/A |
| 21F | Experienced F | 1 | 66/99  *P* < 0.05 * | 76/128  *P* < 0.05 * | 56/103  *P* = 0.431 | N/A |
| 22F | Experienced F | 1 | 35/40  *P* < 0.05 * | 36/53  *P* < 0.05 * | 20/34  *P* = 0.392 | N/A |
| 23F | Experienced F | 1 | 43/60  *P* < 0.05 * | 34/62  *P* = 0.526 | 54/111  *P* = 0.850 | N/A |
| 24F | Experienced F | 1 | 18/22  *P* < 0.05 * | 20/32  *P* = 0.215 | 8/18  *P* = 0.815 | N/A |
| 25F | Experienced F | 1 | 56/80  *P* < 0.05 * | 80/117  *P* < 0.05 * | 50/95  *P* = 0.682 | N/A |
| 26F | Experienced F | 1 | 45/65  *P* < 0.05 * | 99/186  *P* = 0.420 | 111/202  *P* = 0.181 | N/A |
| 27F | Experienced F | 1 | 51/69  *P* < 0.05 * | 30/59  *P* = 1.000 | 22/42  *P* = 0.878 | N/A |
| 28F | Experienced F | 1 | 7/11  *P* = 0.549 | 37/59  *P* = 0.067 | 47/86  *P* = 0.451 | N/A |
| 29F | Experienced F | 1 | 29/41  *P* < 0.05 * | 58/90  *P* < 0.05 * | 43/67  *P* < 0.05 * | N/A |
| 30F | Experienced F | 1 | 30/42  *P* < 0.05 * | 39/76  *P* = 0.909 | 40/78  *P* = 0.910 | N/A |
| 31F | Experienced F | 1 | 43/66  *P* < 0.05 * | 67/109  *P* < 0.05 * | 74/135  *P* = 0.302 | N/A |
| 32F | Experienced F | 1 | 73/101  *P* < 0.05 * | 92/152  *P* < 0.05 * | 11/20  *P* = 0.824 | N/A |
| 33F | Experienced F | 1 | 33/45  *P* < 0.05 * | 27/50  *P* = 0.672 | 38/66  *P* = 0.268 | N/A |
| 34F | Experienced F | 1 | 21/29  *P* < 0.05 * | 27/43  *P* = 0.126 | 31/61  *P* = 1.000 | N/A |
| 35F | Experienced F | 1 | 49/59  *P* < 0.05 * | 33/49  *P* < 0.05 * | 26/53  *P* = 1.000 | N/A |
| 36F | Experienced F | 1 | 27/43  *P* = 0.126 | 53/95  *P* = 0.305 | 51/82  *P* = 0.060 | N/A |
